# Chronic mild stress-induced protein dysregulations correlated with susceptibility and resiliency to depression or anxiety revealed by quantitative proteomics of the rat prefrontal cortex

**DOI:** 10.1038/s41398-021-01267-0

**Published:** 2021-02-24

**Authors:** Wei Liao, Yanchen Liu, Lixiang Wang, Xiao Cai, Hong Xie, Faping Yi, Rongzhong Huang, Chui Fang, Peng Xie, Jian Zhou

**Affiliations:** 1 Institute of Neuroscience, Chongqing Medical University, 400016 Chongqing, China; 2 Basic Medical College, Chongqing Medical University, 400016 Chongqing, China; 3 Shenzhen Wininnovate Bio-Tech Co., Ltd, 410034 Shenzhen, China; 4 Department of Pharmacy, Chongqing Renji Hospital, University of Chinese Academy of Sciences, 400062 Chongqing, China; 5 ChuangXu Institute of Life Science, 400016 Chongqing, China

**Keywords:** Depression, Molecular neuroscience

## Abstract

Chronic stress is a significant risk factor for depression as well as anxiety disorders. Yet, the stress-induced specific and common molecular dysregulations of these disorders have not been fully understood. Previously, we constructed a chronic mild stress (CMS) rat model to separate and obtain depression-susceptible, anxiety-susceptible, and insusceptible groups. In this study, the prefrontal cortical proteomes of the three stressed groups were comparatively profiled utilizing isobaric tags for relative and absolute quantitation (iTRAQ)-coupled tandem mass spectrometry approach. A total of 212 protein dysregulations were identified, potentially correlating to susceptibility or resilience to CMS-induced depression or anxiety, and thus might serve as potential protein targets for further investigation. In addition, independent analysis by parallel reaction monitoring identified changes in Gfap, Rhog, Gnai2, Ppp1r1b, and Uqcrh; Tubb6, Urod, Cul1, Spred1, and Gpcpd1; Acadl, Ppp1r1a, Grm2, Mtor, Lsm8, Cplx2, and Tsta3 that were distinctly correlated to depression-susceptible, anxiety-susceptible, or insusceptible groups, respectively. This suggested that identical CMS had different effects on the protein regulation system of the rat prefrontal cortex. Collectively, the present proteomics study of the prefrontal cortex established a significant molecular basis and offered new insights into the specificity and commonality of pathophysiologic mechanisms underlying susceptibility and resiliency to stress-induced depression or anxiety.

## Introduction

Depression and anxiety, as two chronic and common psychiatric disorders, have a negative influence on socio-occupational factors and well-being of patients, families, and society^1–3^. Many studies demonstrate that depression and anxiety have common risk factors, including chronic stress and life event stress^4–6^. Mounting evidence indicates that chronic stressful life events provide the adverse environmental factors underlying the depression and anxiety etiologies^7,8^. Yet, many individuals do not exhibit anxious and depressive symptoms even after exposure to chronic stress^9–12^. Chronic mild stress (CMS) has been widely used to induce depressive and anxious behaviors in rodents to model the environmental risk factors influencing humans^7,10^. To illustrate the potential biological etiology and pathophysiology of depression and anxiety, it is meaningful to focus on the neural substrates underlying susceptibility and resiliency to these stress-induced disorders^9^.

In general, depression and anxiety clinically display distinct fundamental symptoms, but are often diagnosed simultaneously^13,14^. Because of this considerable overlapping in comorbidity and pathophysiology, the data in most clinical and basic studies are often mixed^8,15^, potentially obscuring our understanding of the factors modulating the two diseases. Several researchers are slowly starting to analyze separately non-comorbid subjects to reveal the unique and common characteristics of the nervous systems^16–19^. Both depression and anxiety are heterogeneous diseases controlled through various brain structures, including the hippocampus and prefrontal cortex. Several studies have suggested that depression and anxiety result in a reduced volume and dendritic spine density of the prefrontal cortical regions in humans and animals^6,12^. The prefrontal cortex, as a sensitive brain region to stress, participates in executive, cognitive, and socio-emotional functions^20^. Chronic stress-induced morphological and functional changes that take place in the prefrontal cortex can result in both the cognitive and emotional dysfunctions leading to depression or anxiety^12,21^. Despite the abnormal prefrontal cortical plasticity observed in depressive, as well as anxious individuals, the corresponding inherent patterns might be markedly distinct and remain unknown. Hence, to identify distinct and common molecular features underlying susceptibility and resilience to depression or anxiety has become a pressing need.

In our previous research, hippocampal quantitative proteome profiling was used to explore distinct and common proteins related to CMS-induced depression- and anxiety-like behaviors^22^. Based on the behavioral testing data, the depression-susceptible, anxiety-susceptible, and insusceptible groups were obtained to be used as the three different CMS responses. In the present study, the prefrontal cortical tissues from this sample of CMS model rats^22^ were utilized to further explore stress-induced depression and anxiety disorders. The proteomes of the prefrontal cortex from the depression-susceptible, anxiety-susceptible, insusceptible, and control groups were comparatively profiled, providing the molecular basis correlated to adaptive and maladaptive phenotypes of depression or anxiety. Our present results can provide a window to understand the specific and common molecular mechanisms that underlie stress-induced depression or anxiety and stress resiliency.

## Materials and methods

### Animals

Male Sprague–Dawley albino rats weighing ~250 g were acquired from the Animal Center of Chongqing Medical University (China). The animal protocols of this study were approved by the local Ethics Committee. These animals were cared for following the National Institutes of Health protocols for the use and care of laboratory animals. All animals were individually housed and maintained on a 12 h light/dark cycle with temperatures from 21 to 22 °C, and relative humidity, 55 ± 5%. The animals were allowed to have access to food and water ad libitum.

### CMS exposure to rats

The animals were habituated to sucrose consumption and the CMS procedure was carried out, as described previously^22^. Based on baseline sucrose intake from the three tests, the rats were divided randomly among the stressed or control units. The stressed sample was subjected to CMS for 8 weeks, while the control group remained without intervention. Paired housing, a soiled cage, a 45° cage tilt, water scarcity, strobe light, continuous lighting, an empty water bottle, and white noise were used to form the CMS model.

### Behavioral testing

As previously described^22^, we conducted a sucrose preference test (SPT) to measure the rat depression-like behavior (anhedonia). The behavioral-based formula was used to assess sucrose preference: sucrose preference = (sucrose intake/total fluid intake) × 100%. Afterward, a forced swimming test (FST) was conducted to gauge despair-like behavior, as formerly illustrated^22^. The behaviors of the rats were determined in a water-filled Plexiglas cylinder and recorded using a video monitoring system. Meanwhile, the anxiety-like behavior of rats was evaluated by an elevated plus-maze test (EMT), as previously described^22^. During the CMS process, the rat body weights were recorded every week.

### Collection of tissue and extraction of proteins

After the behavioral testing, these rats underwent decapitation, and their entire brains were quickly excised. The prefrontal cortex tissues were separated from the brain and frozen quickly in liquid nitrogen while housed at −80 °C. For the extraction of proteins, the tissues were homogenized in an SDT lysis buffer containing 4% SDS, 100 mM dithiothreitol, 100 mM Tris–HCl, pH 8.0, and protease inhibitors. These protein extracts were heated at 100 °C for five minutes and then centrifuged for 10 min at 40,000 × *g*. Pierce bicinchoninic acid assay was performed to measure the protein concentrations in the supernatant.

### Filter-aided sample preparation (FASP)-based digestion of proteins

As per our previously described protocol^23^, the FASP-based method with 10-kD ultrafiltration centrifuge tubes was employed for protein digestion. Briefly, the lysates were diluted using urea buffer (150 mM Tris–HCl, pH 8.0, 8 M urea). The denatured proteins were alkylated with 50 mM iodoacetamide for 30 min in the dark. After centrifugation, the proteins were washed two times using the urea buffer. Subsequently, the proteins were digested overnight at 37 °C with trypsin. The resulting digests were collected by repeated centrifugation and washing. The peptide solution was then dried in a Speed Vac.

### iTRAQ labeling and high-pH reversed-phase liquid chromatography (RPLC) fractionation

Peptides from the FASP-based digestion were labeled with 8-plex isobaric tags for relative and absolute quantitation (iTRAQ) reagents following the instructions of the manufacturer. Eight samples from the control and the stressed groups were labeled using the reagents 113–121. Each sample was obtained from two or three rats in each group. After peptide labeling, these samples were mixed and subsequently fractionated by offline high-pH RPLC, where the peptides were fractionated via an 80-min linear gradient elution of 5–38% high-pH buffer (10 mM ammonium formate, 90% acetonitrile, pH 10.0). The flow rate was 0.3 ml/min. The acquired 16 fractions were desalted and lyophilized for the following analysis.

### Liquid chromatography-tandem mass spectrometry

The lyophilized peptides were reconstituted in 0.1% formic acid, and loaded into a nanoViper C18 trap column (3 μm, 100 Å). The online chromatographic separation was performed on Thermo Scientific Easy-nLC 1200 system. Post trapped and desalted with 100% solution A (0.1% formic acid), the peptides were separated with solution B (80% acetonitrile/0.1% formic acid) elution gradient from 8 to 38% in 50 min on an analytical column (50 μm × 150 mm, 3 μm C18 100 Å). Mass spectrometry (MS) experiment was performed on a ThermoFisher Q-Exactive Orbitrap mass spectrometer fitted with a Nano Flex ion source. The MS was operated using an ion spray voltage of 1.9 kV and an interface heater temperature of 275 °C. A data-dependent acquisition mode with full MS scans was used to acquire tandem MS data. MS1 spectra were acquired in the *m*/*z* range of 350–1200, and MS2 spectra were acquired in the *m*/*z* range of 110–1200. The acquisition survey scans of 250 ms were followed by up to 14 product ion scans of 50 ms. The MS spectra with charge state 2 and above were selected for fragmentation by higher-energy collision dissociation. The dynamic exclusion time was set for 25 s.

### Data analysis

Proteome Discoverer (v2.1, ThermoFisher) software and the Sequest HT search engine were used to identify and quantify peptides and proteins based on the UniProt_Rat release 2017_12 database. The search parameters allowed for trypsin missed cleavages of two, 10 p.p.m. precursor fragment mass tolerance, and 0.05 Da fragment mass tolerance. Fixed modifications were set for iTRAQ 8-plex on N-term and Lys and Carbamidomethylation on Cys. Variable modification settings included oxidation on Met, acetylation on protein N-term, deamidation on Asn and Gln, and Pyro-Glu. For identification of proteins and peptides, the peptide-spectrum-match false discovery rate was controlled <1.0% with the Percolator module, as previously described^24^. The relative ratios of peptides and proteins across samples were computed and quantified using Reporter Ions Quantifier and Peptide and Protein Quantifier nodes. This data was imported into Microsoft Excel for manual interpretation. Afterward, a two-tailed Student’s *t* test was performed on the ratios of the identified proteins. The proteins with a 1.2-fold change and *p* < 0.05 were considered to be significantly differentially expressed. The raw data have been deposited to the ProteomeXchange Consortium (http://proteomecentral.proteomexchange.org) via the iProX partner repository with the dataset identifier PXD022045 (ref. ^25^).

### Functional and network analysis

We used the OmicsBean tool (http://www.omicsbean.cn/) for enrichments of biological processes (BP), molecular functions (MF), and cellular components (CC) based on Gene Ontology (GO, http://www.geneontology.org/) terms^22,26^. Kyoto Encyclopedia of Genes and Genomes (KEGG, http://www.genome.jp/kegg/) analysis was conducted for pathway enrichment, with a *p* value of <0.1 considered significant, as previously described^27^. Furthermore, protein–protein interaction (PPI) was assessed using the database of the Search Tool for the Retrieval of Interacting Genes/Proteins (STRING) under the medium confidence (STRING score = 0.4), and the yielded network was visualized with Cytoscape tool, as reported previously^22^. To explore the drug relevance of these differential proteins, we performed a phenotype-target-drug network analysis. The antidepression and antianxiety drugs and their primary targets were searched and collected from the DrugBank database (Version 5.1.4; https://www.drugbank.ca/). Species type for these target proteins was converted from *Homo sapiens* to rattus norvegicus. Through the use of the STRING detection, the interactions between the differential proteins and the target proteins were obtained. To construct the phenotype-target-drug network, the direct interactions were further mapped to the corresponding drugs using Cytoscape software.

### Parallel reaction monitoring (PRM) analysis

Protein extraction and digestion were conducted following the above iTRAQ experiment. The tryptic peptides were placed in the mass spectrometer. Peptide fragmentation was performed with a normalized collision energy of 28. The fragments were identified at a 35,000 resolution in the Orbitrap. The acquired raw data were then analyzed with the Proteome Discoverer. The resulting MS data were processed with Skyline software (v19.1).

### Statistical analysis

The statistical analysis was conducted with SPSS software. All the results were analyzed using Student’s *t* test and expressed as means ± standard error. Data were considered to be statistically significant at *p* values <0.05.

## Results

### Behavioral evaluation of the CMS model rats

In general, the preclinical model offered by the CMS protocol can help to explore potential molecular features of depression and anxiety disorders. In this study, SPT and FST were used to assess the CMS-induced depression-like behavior (anhedonia and behavioral despair). Afterward, the EMT experiment was used to index anxiety-like behavior. Given the rat prefrontal cortical sample used was from the same batch as the CMS model used in our previous publication^22^, it was briefly illustrated that the sucrose inclination of the depression-susceptible group was diminished, whereas the immobility time was increased when compared to the other groups. Despite this, some stressed rats did not show depression-like behavior, yet their anxiety-like behavior was found by the EMT. Accordingly, these stressed rats were classified as the anxiety-susceptible group. Also, there were no significant differences in the SPT, FST, or EMT between the insusceptible and control groups. This suggests the insusceptible group did not include any anxiety-like or depression-like phenotypes. By these evaluations, we finally obtained a subset of the depression-susceptible, anxiety-susceptible, insusceptible, and control groups. These results demonstrated that the CMS protocol offered an effective way of defining CMS-induced molecular patterns related to susceptibility and resiliency to depression or anxiety.

### Comparative analysis of the rat prefrontal cortical proteome

**Fig. 1:**
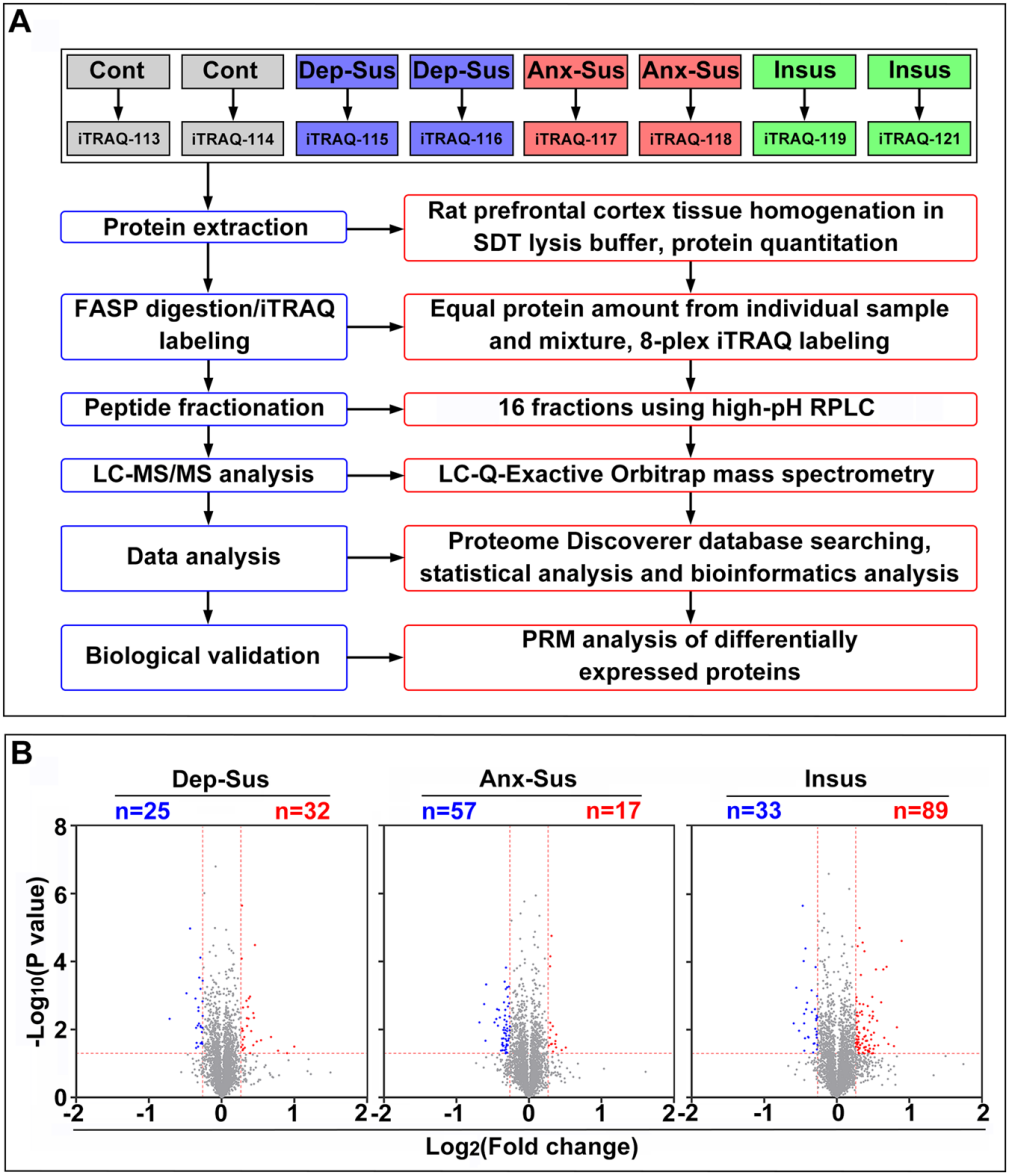
Comparative profiling of the prefrontal cortical proteomes of the stressed rats. **A** Analysis flowchart of isobaric tags for relative and absolute quantitation (iTRAQ)-based quantitative proteomics of the prefrontal cortex tissue samples from the control (Cont), depression-susceptible (Dep-Sus), anxiety-susceptible (Anx-Sus), and insusceptible (Insus) groups. **B** Identification of differentially expressed proteins in the prefrontal cortex. Volcano plot displaying protein expression variations in the Dep-Sus, Anx-Sus, and Insus groups. The *x*-axis shows the average fold change (log2), and the *y*-axis shows the negative *p* value (log10). Low and high relative expressions were indicated in blue and red, respectively.

According to our previously established quantitative strategy^22^, expression alterations in the prefrontal cortical proteome of the stressed rats were comparatively analyzed. A workflow of the iTRAQ-based quantitative approach is illustrated in Fig. 1A. Comparative proteome analysis was conducted on five animals per group. As described previously, the prefrontal cortex tissue samples taken from two to three rats in each group were mixed in an equal protein amount from the individuals^28^. In our present study, a total of 3604 nonredundant proteins were quantified as below 1% FDR (Supplementary Table S1). Through comparatively profiling the prefrontal cortical proteomes of the depression-susceptible, anxiety-susceptible, insusceptible, and control groups, 212 stress-responsive proteins were identified with >1.2-fold changes and *p* values <0.05 (Supplementary Table S1).

### Functional and network analysis of differentially expressed protein in the stressed rats

**Fig. 2:**
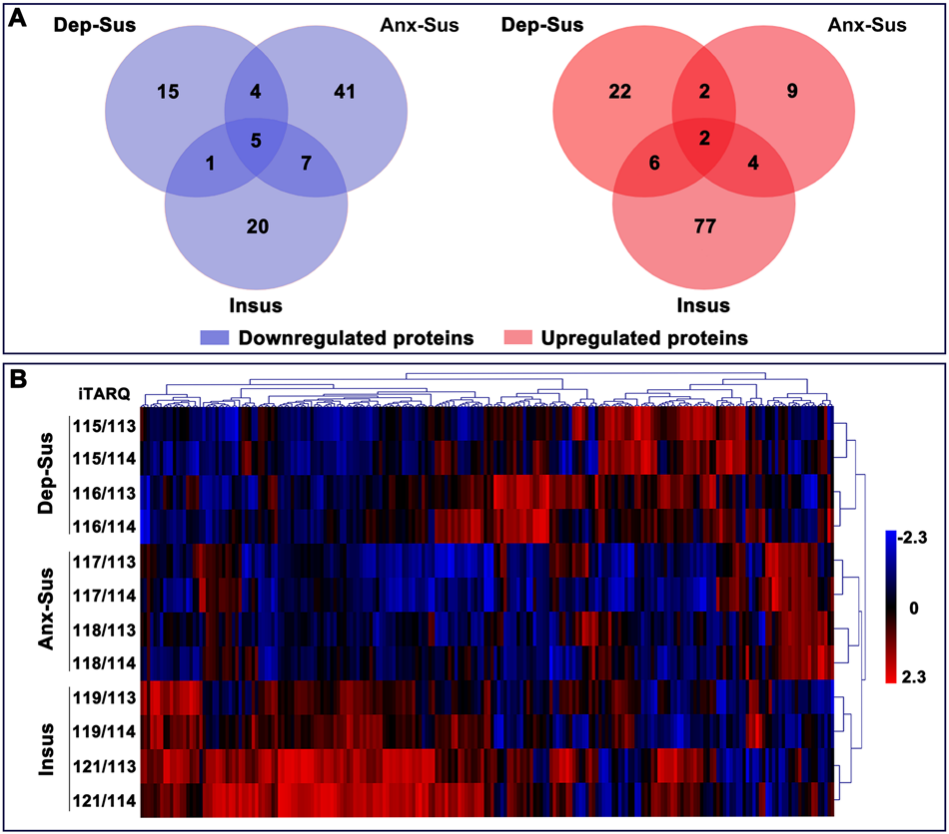
Expression analysis of the identified differentially expressed proteins. **A** Venn diagrams displaying the differential protein number among the depression-susceptible (Dep-Sus), anxiety-susceptible (Anx-Sus), and insusceptible (Insus) groups. **B** Clustering of differential proteins from the three groups using a visualized heatmap. Lower expressions were shown by blue and higher by red. Various color intensities indicated the expression levels and a log2 scale was used in the color bar. The included eight labels were indicated on the *y*-axis.

Comparative proteome profiling in the prefrontal cortex of the CMS-treated rats revealed 25 downregulated and 32 upregulated proteins in the depression-susceptible group, 57 downregulated and 17 upregulated proteins in the anxiety-susceptible group, and 33 downregulated and 89 upregulated in the insusceptible group. In the two CMS-susceptible groups, just 13 proteins were observed to be regulated in the same way, which might represent some shared aberrant elements between depression and anxiety disorders. Also, 25 similarly regulated proteins were found among the two susceptible and the insusceptible groups as a CMS-exposed result (Fig. 2A). Intriguingly, up to 87% of these proteins were distinctly associated with the three phenotypes. This suggested that these stressed rats had distinct molecular patterns as responsive to CMS. Further analysis by unsupervised hierarchical clustering of the 212 differential proteins classified these samples into three different cohorts, also reflecting the three distinct responses to stress (Fig. 2B).

**Fig. 3:**
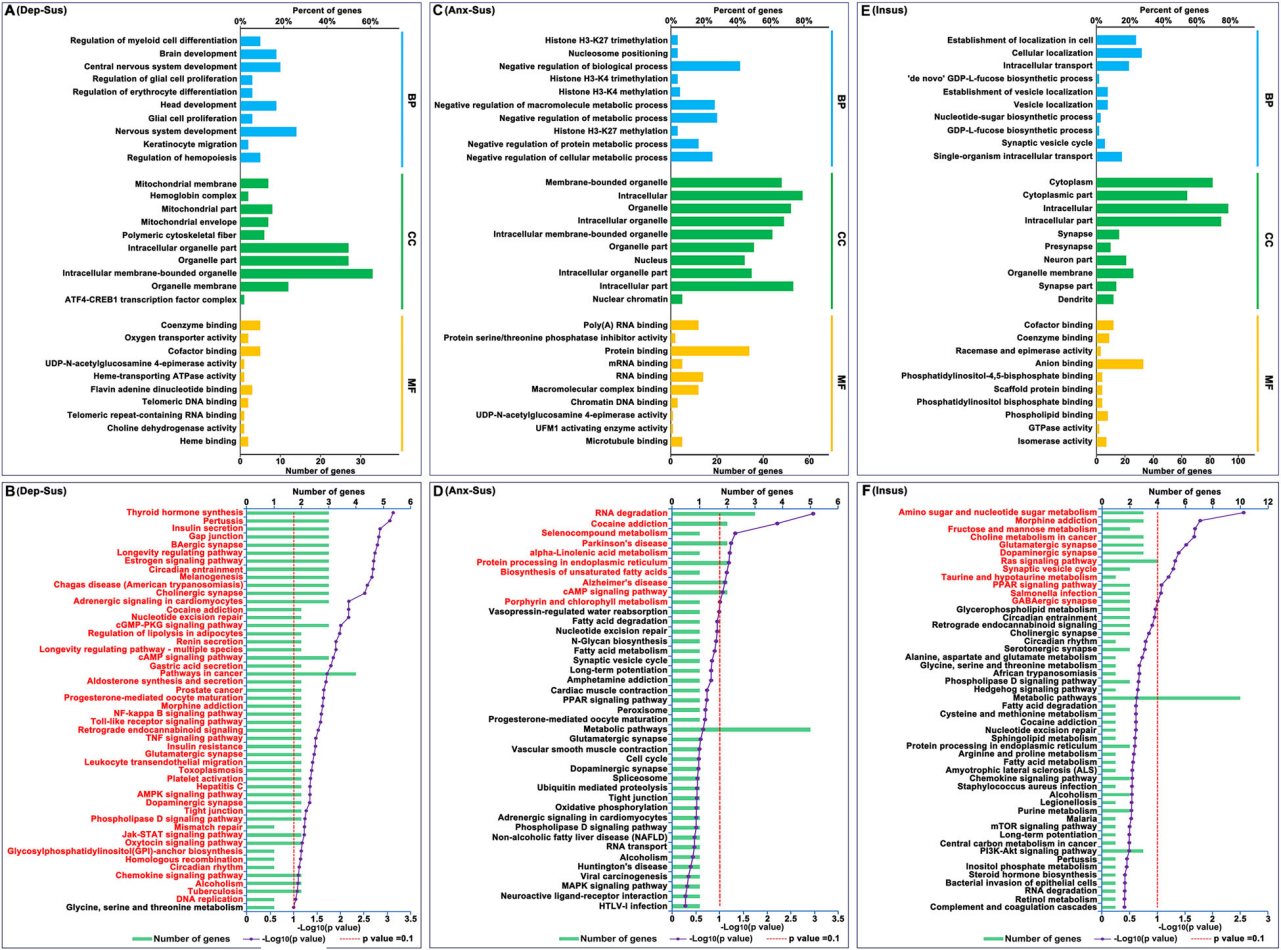
Enrichment analysis of Gene Ontology and Kyoto Encyclopedia of Genes and Genomes pathway terms. Differentially expressed proteins from the depression-susceptible (Dep-Sus, **A**), anxiety-susceptible (Anx-Sus, **C**), and insusceptible (Insus, **E**) groups were analyzed with Gene Ontology (GO) database, and the top ten enriched biological process (BP), cellular component (CC), and molecular function (MF) terms were shown. The significantly enriched Kyoto Encyclopedia of Genes and Genomes (KEGG) pathway terms in the Dep-Sus (**B**), Anx-Sus (**D**), and Insus (**F**) groups were indicated with titles in red. The *x*-axis displays the negative *p* value (log10).

To deeply understand the significant biological functions and pathways associated with behavioral phenotypes, these differential proteins from the three groups were investigated using the OmicsBean tool. The 57 differentially expressed proteins in the depression-susceptible group were analyzed based on the GO and KEGG pathway databases. In total 439, 66, 88, and 49 terms in the BP, CC, MF, and KEGG pathway categories were significantly enriched (Supplementary Table S2). The ten leading enriched GO terms are illustrated in Fig. 3A. BP category analysis demonstrated that many proteins were related to the nervous system and brain development, and erythrocyte and myeloid/glial cell proliferation. Most proteins in the CC category were observed to belong to mitochondrial and organelle parts. MF category analysis indicated that a majority of proteins were involved in protein, DNA and RNA binding, and enzyme activity. The KEGG pathway analysis revealed the dysregulated proteins mainly participated in various signaling, secretion, and synapse pathways (Fig. 3B).

Meanwhile, GO and KEGG pathway enrichment of the 74 dysregulated proteins in the anxiety-susceptible group were analyzed. A total of 407 BP, 105 CC, 83 MF, and 10 KEGG pathway terms were significantly enriched. The top ten enriched GO terms are shown in Fig. 3C. BP category analysis indicated that most proteins were related to histone trimethylation and methylation and metabolic processes. CC category analysis indicated the differential proteins belonging to the nucleus, intracellular and organelle components, and MF indicated the majority of the proteins were predicted to be engaged in RNA, protein, and complex binding, as well as enzyme activity. Enrichment analysis of KEGG pathways showed the dysregulated proteins were mainly involved in various metabolic and neuropsychiatric disease pathways (Fig. 3D).

Afterward, we also conducted GO and KEGG pathway enrichment of the 122 differential proteins identified in the insusceptible group. A total of 410 BP, 113 CC, 130 MF, and 12 KEGG pathway terms were significantly enriched. The top ten enriched GO terms are displayed in Fig. 3E. BP category analysis signaled that the majority of proteins were engaged in cellular and vesicle localization, intracellular transport, biosynthetic process, and synaptic vesicle cycle, and CC displayed that most proteins were located in the cytoplasmic, intracellular, neuron, and synapse parts. The majority of proteins in the MF category were predicted to be involved in cofactor, coenzyme, scaffold protein, and small molecule binding, as well as enzyme activity, and KEGG pathway enrichment revealed that the dysregulated proteins were primarily implicated in various metabolism, synapse, and signaling pathways (Fig. 3F).

**Fig. 4:**
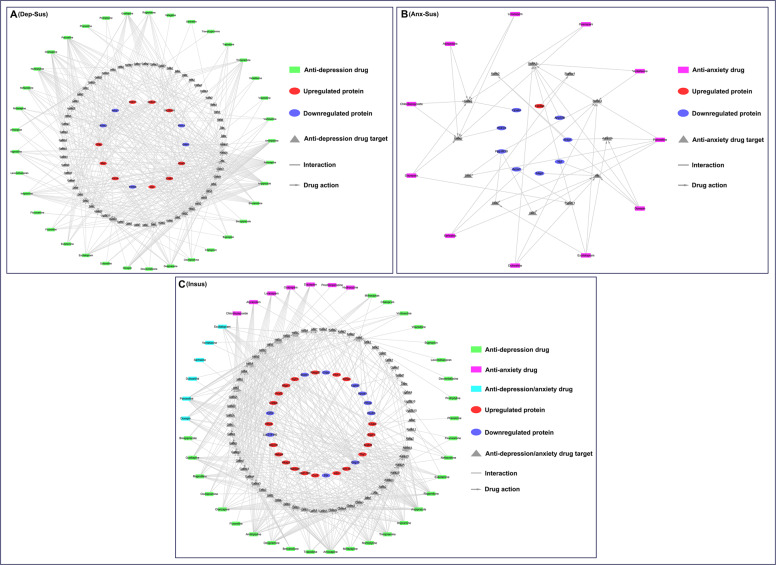
Analysis of phenotype-target-drug networks. The networks from the depression-susceptible (Dep-Sus, **A**), anxiety-susceptible (Anx-Sus, **B**), and insusceptible (Insus, **C**) groups were built by the Search Tool for the Retrieval of Interacting Genes/Proteins (STRING) and Cytoscape.

Further, we carried out phenotype-target-drug network analysis to explore the drug relevance of the differential proteins. A total of 38 antidepression and 13 antianxiety drugs approved by the US FDA were firstly retrieved through searching DrugBank database. Details of these drugs, including drug name, DrugBank ID, and target proteins, are shown in Supplementary Table S3. A total of 113 primary target proteins were obtained from 38 antidepression drugs, and a total of 70 primary target proteins were obtained from 13 antianxiety drugs. After species type was converted from human to rat, the target proteins together with the differential proteins were introduced into the STRING database to obtain their interactions. The direct interactions with medium confidence were then mapped to the corresponding drugs in Cytoscape for the construction of the phenotype-target-drug networks (Fig. 4). In the networks, the primary target proteins acted as a link between the differential proteins and the drugs. The results showed that 14 differential proteins from the depression-susceptible group were correlated with 37 antidepression drugs. Meanwhile, 9 differential proteins from the anxiety-susceptible group were found to be correlated with 11 antianxiety drugs, and 30 differential proteins from the insusceptible group were correlated with 42 antidepression and/or antianxiety drugs. By means of these functional links, the 52 phenotype-associated proteins could be predicted to be potential secondary targets of the antidepression/anxiety drugs.

**Fig. 5:**
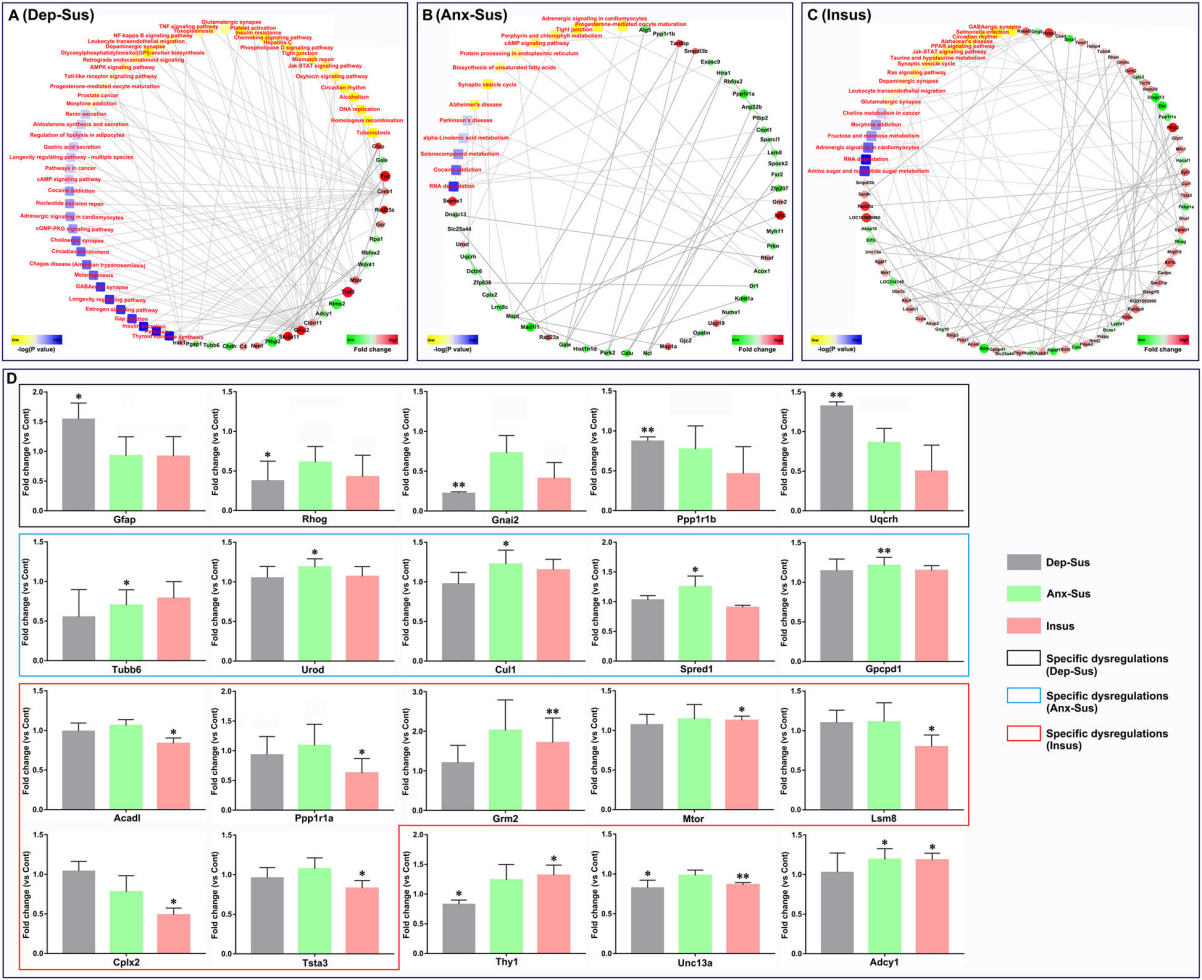
Protein–protein interaction and parallel reaction monitoring analyses of the differentially expressed proteins. The protein–protein interaction (PPI) networks of the three groups (depression-susceptible (Dep-Sus, **A**), anxiety-susceptible (Anx-Sus, **B**), and insusceptible (Insus, **C**)) were constructed based on protein alterations and Kyoto Encyclopedia of Genes and Genomes (KEGG) pathway term enrichments. Proteins/genes were represented by circular nodes. The KEGG pathway is indicated by the rectangle, shaded based on the *p* value. Higher *p* value is shown in yellow and lower *p* value in blue. **D** Parallel reaction monitoring (PRM) analysis of the differentially expressed proteins in the Dep-Sus, Anx-Sus, and Insus groups relative to the control (Cont). Gfap, Rhog, Gnai2, Ppp1r1b, Uqcrh, Tubb6, Urod, Cul1, Spred1, Gpcpd1, Acadl, Ppp1r1a, Grm2, Mtor, Lsm8, Cplx2, Tsta3, Thy1, Unc13a, and Adcy1 were detected on the prefrontal cortical protein extracts of the rats; *n* = 4–5, *p < 0.05, **p < 0.01.

Subsequently, we focused on the proteomics-inferred PPI networks in the three stressed groups (Fig. 5A–C). PPI networks of the three groups were constructed by the use of those differential proteins correlated to the significantly enriched KEGG pathways. Based on a unified conceptual framework, 23, 46, and 69 proteins as key nodes were identified from the three networks generated in the depression-susceptible, anxiety-susceptible, and insusceptible groups, respectively. The included PPI networks revealed a similar correlation between the dysregulated proteins and the KEGG pathways, while offering a minor interactome pool associated with the three different behavioral phenotypes.

### PRM analysis of CMS-related proteins

In the present study, 20 differential proteins of interest from the significantly enriched KEGG pathways and networks were further independently validated by the PRM analysis. Overall, these PRM results mirrored the data from the iTRAQ-based experiment (Supplementary Fig. S1). Similar to other proteomics studies^29–32^, there were some discrepancies between the two quantitative data sets. Besides the detection difference of the two methods, the additional pooling step in the iTRAQ-based analysis workflow might be another cause^29^. As shown in Fig. 5D, the expressions of Gfap and Uqcrh were significantly upregulated, whereas Rhog, Gnai2, and Ppp1r1b were downregulated in the depression-susceptible group when contrasted to the control group. It could be observed that the expressions of Urod, Cul1, Spred1, and Gpcpd1 were significantly upregulated, while Tubb6 was downregulated in the anxiety-susceptible group compared with the control group. Moreover, we found that the expressions of Grm2 and Mtor were significantly upregulated while Acadl, Ppp1r1a, Lsm8, Cplx2, and Tsta3 were downregulated among the insusceptible group when contrasted with the control group. In addition, the expression level of Thy1 was shown to be significantly downregulated among the depression-susceptible group, whereas it was upregulated in the insusceptible group compared with the control group. The reduced level of Unc13a in both the depression-susceptible and insusceptible groups, and the elevated level of Adcy1 in both the anxiety-susceptible and insusceptible groups were recognized when compared with the control group.

## Discussion

In depression and anxiety disorders, chronic stress is generally believed to be an adverse risk factor. Accordingly, researchers utilized the CMS protocol to generate depressive and anxious behaviors in rats^7,10^. In our previously established CMS model, the three different phenotypes (depression-susceptible, anxiety-susceptible, and insusceptible) of the stressed rats have been identified based on behavioral testing^22^. This model offered a valuable way for the identification of unique and common molecular features of vulnerability and resiliency to depression or anxiety, thereby contributing to translational biomedical research. Investigating the protein expression profiles linked to the three different behavioral phenotypes can offer new insights into our knowledge of clinical anxiety and depression disorders.

To reveal the phenotype-associated protein dysregulations, herein the prefrontal cortical proteomes of the stressed rats were comparatively profiled using an iTRAQ-based quantitative approach. Compared with the unstressed controls, 212 differentially expressed proteins were identified in the prefrontal cortex of depression-susceptible, anxiety-susceptible, and insusceptible rats. The overlapping protein expression changes between the two susceptible groups potentially represented the shared molecular patterns of depression and anxiety disorders. Those similar dysregulations between the insusceptible and anxiety/depression-susceptible groups were considered as general CMS-responsive protein alterations. Interestingly, the distinctly dysregulated proteins in the three stressed groups might represent the differences between the CMS-induced behavioral phenotypes. The clustering analysis also demonstrated that the three CMS-exposed cohorts exhibited distinctive protein dysfunctional profiles. These proteomics data would facilitate studies to uncover protein systems, and pathways related to susceptibility and resilience to stress-induced anxiety or depression.

Our proteomics and bioinformatics analyses suggested affected pathways in the prefrontal cortex specifically correlated to CMS-induced behavioral phenotypes of resilience, depression, and anxiety. The pathway analysis showed that the differentially expressed proteins were significantly enriched for signaling, secretion, and synapse dysfunctions in the depression-susceptible group, metabolism and neuropsychiatric disease-associated dysregulations in the anxiety-susceptible group, and metabolism, synapse, and signaling repercussions in the insusceptible group. Subsequently, the network analysis unraveled the protein dysregulation systems corresponding to these significantly enriched pathways and potentially provided some valuable information for the pathophysiological basis of the three stress-induced behavioral phenotypes. Furthermore, we further explore the drug relevance of these differential proteins through the use of phenotype-target-drug network analysis. As a result, the 52 phenotype-associated proteins were identified to be correlated with the antidepression/anxiety drugs. It can be assumed that the drugs may act on the potential primary targets to affect expressions of the correlated differential proteins, thereby ameliorating depression and anxiety symptoms in stress-susceptible subjects or enabling them to resist stress. Accordingly, these 52 differential proteins may represent new important secondary targets that are worthy of further study for the drug development in depression and anxiety disorders.

In the present study, the 20 differentially expressed proteins involved in the significant KEGG pathways were further independently validated using the PRM-based approach. The results showed that Gfap, Rhog, Gnai2, Ppp1r1b, and Uqcrh were specifically dysregulated in the prefrontal cortex of the depression-susceptible group, while Tubb6, Urod, Cul1, Spred1, and Gpcpd1 were specifically dysregulated in the anxiety-susceptible group. The molecular response specificity in the prefrontal cortex suggested that identical CMS could differently impact the MF, thereby potentially pushing forward various BP and correlated rat behavioral phenotypes. Interestingly, we found that Acadl, Ppp1r1a, Grm2, Mtor, Lsm8, Cplx2, and Tsta3 were specifically dysregulated in the insusceptible group. This suggests a potential pathway to coping with the CMS-induced protein dysfunctions in the prefrontal cortex. It could be speculated that these distinct changes might be involved in positive molecular mechanisms of stress protection in the rat prefrontal cortex^9–12^. Furthermore, we also noted the opposite change of Thy1 in depression-susceptible and insusceptible groups, which might be an important clue for resilience to depression.

These PRM-detected phenotype-specific dysregulated proteins were primarily involved in the synapse, signaling, and metabolic pathways. Cplx2, Gnai2, and Grm2 were involved in synaptic plasticity-associated pathways, such as synaptic vesicle cycle and glutamatergic synapse. Gfap, Spred1, and Mtor participated in the Jak-STAT signaling pathway, and Urod, Gpcpd1, and Tsta3 were involved in various metabolic processes, such as porphyrin and chlorophyll, choline, and sugar metabolisms. Previously, these important pathways have been preliminarily implicated in stress-related disorders^5,6^. In the present study, the found protein expression perturbations distinctly associated with the three behavioral phenotypes could provide clearer information for future research.

However, there are several notable limitations of this study. First, although protein dysregulations in the prefrontal cortex of the stressed rats were identified, the precise functions and roles of these proteins behind such aberrations need to be further explored to obtain a better understanding of the underlying mechanisms of stress resiliency and stress-induced depression/anxiety. Second, it is important to further investigate the expressions of these phenotype-associated differential proteins in the prefrontal cortex tissues of patients with depression and anxiety for translational research.

## Conclusion

In summary, we utilized the iTRAQ-based quantitative proteomics method to outline the consequences of CMS on the rat prefrontal cortex proteome. The unbiased profiles detected several prefrontal cortical protein candidates that were potentially correlated with susceptibility and resiliency to stress-induced anxiety or depression, and thereby provided new insights into the protein dysregulation mechanisms behind CMS in the stressed rats. The present results could serve as an important molecular basis, and enhance our understanding of the physio-pathological differences and similarities of stress-induced depression/anxiety and stress resistance.

## Supplementary information

Supplementary information

Supplementary Table S1

Supplementary Table S2

Supplementary Table S3
