# Supplementary material for: Chronic mild stress-induced protein dysregulations correlated with susceptibility and resiliency to depression or anxiety revealed by quantitative proteomics of the rat prefrontal cortex
Source: Transl Psychiatry. 2021 Feb 24;11:143. doi: 10.1038/s41398-021-01267-0 (PMC7904772; doi:10.1038/s41398-021-01267-0)
Supplement: Supplementary file 1 — Supplementary information [file 41398_2021_1267_MOESM1_ESM.docx]

**Supplementary information**

**Chronic mild stress-induced protein dysregulations correlated with susceptibility and resiliency to depression or anxiety revealed by quantitative proteomics of the rat prefrontal cortex**

Wei Liao^1,2^, Yanchen Liu^1,2^, Lixiang Wang^3^, Xiao Cai^1,2^, Hong Xie^1,4^, Faping Yi^1,2^, Rongzhong Huang^5^, Chui Fang^3,^*, Peng Xie^1,2,^*, Jian Zhou^1,2,^*

^1^Institute of Neuroscience, Chongqing Medical University, Chongqing 400016, China

^2^Basic Medical College, Chongqing Medical University, Chongqing 400016, China

^3^Shenzhen Wininnovate Bio-Tech Co., Ltd, Shenzhen 410034, China

^4^Department of Pharmacy, Chongqing Renji Hospital, University of Chinese Academy of Sciences, Chongqing 400062, China

^5^ChuangXu Institute of Life Science, Chongqing 400016, China

These authors contributed equally: Wei Liao, Yanchen Liu, Lixiang Wang

*To whom correspondence should be addressed:

Peng Xie and Jian Zhou

Institute of Neuroscience, Chongqing Medical University, 1 Yixueyuan Road, Yuzhong District, Chongqing 400016, China. Tel: +86-23-68485763. E-mail addresses: xiepeng@cqmu.edu.cn (P. Xie), zhoujian@cqmu.edu.cn (J. Zhou).

Chui Fang

Shenzhen Wininnovate Bio-Tech Co., Ltd, Nanshan Yungu Innovation Industrial Park, Taoyuan Street, Nanshan District, Shenzhen 410034, China. E-mail address: fancy5029@126.com (C. Fang).

**Supplementary information**

**Supplementary Figure S1.** Comparison between isobaric tags for relative and absolute quantitation (iTRAQ)-based and parallel reaction monitoring (PRM)-based results in this study. Dep-Sus, depression-susceptible; Anx-Sus, anxiety-susceptible; Insus, insusceptible; Cont, control.

**Supplementary Table S1.** Complete protein list identified from the depression-susceptible (Dep-Sus), anxiety-susceptible (Anx-Sus), and insusceptible (Insus) groups using the isobaric tag for relative and absolute quantitation (iTRAQ)-based proteomic approach. The differentially-expressed proteins were highlighted in grey.

**Supplementary Table S2.** Complete enrichment list of gene ontology (GO) biological process (BP), cellular component (CC), molecular function (MF), and Kyoto Encyclopedia of Genes and Genomes (KEGG) pathway terms of the differentially-expressed proteins from the depression-susceptible (Dep-Sus), anxiety-susceptible (Anx-Sus), and insusceptible (Insus) groups. The significantly-enriched terms were indicated in grey.

**Supplementary Table S3.** Primary target proteins of FDA-approved anti-depression and anti-anxiety drugs.


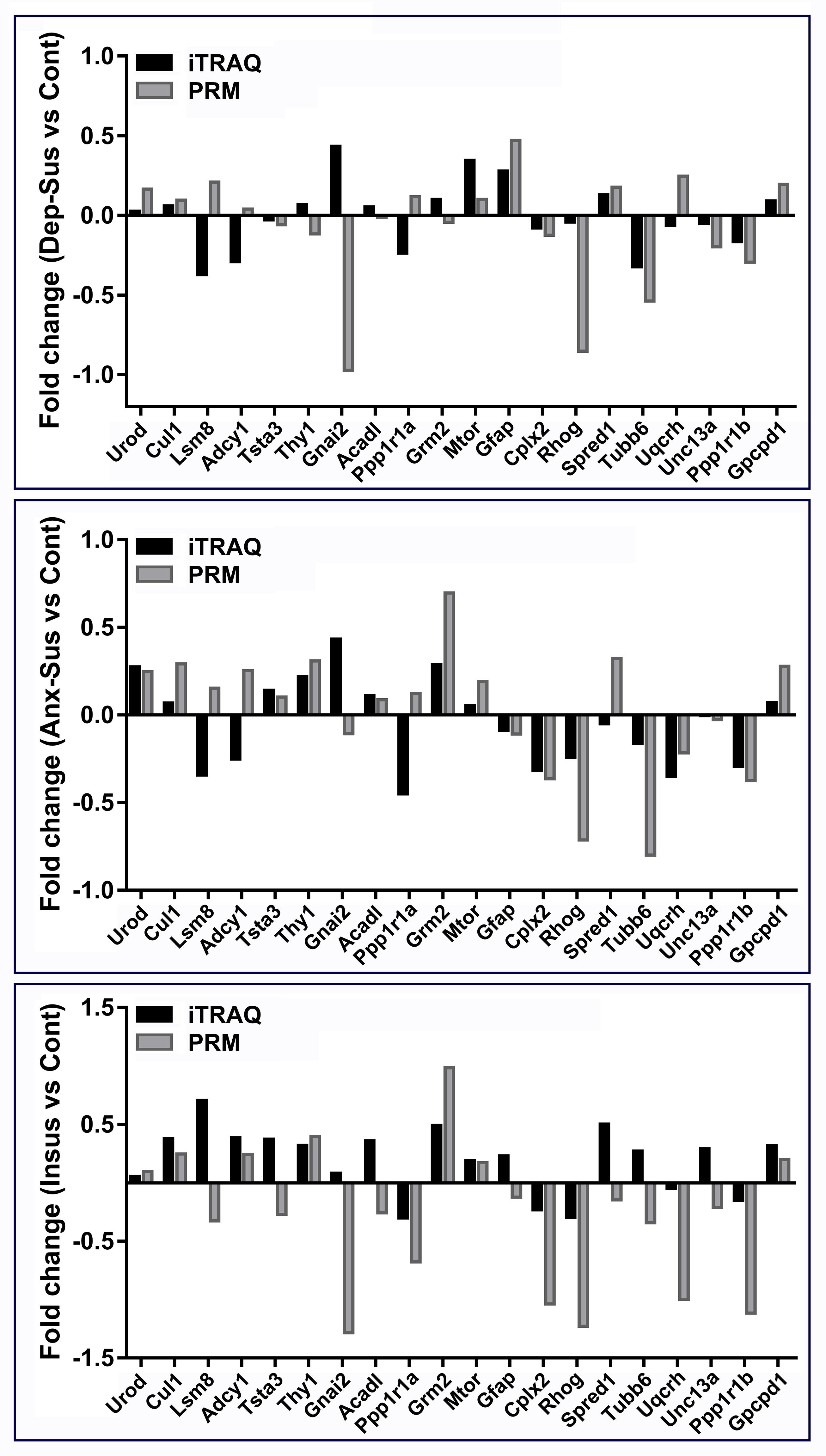


**Supplementary Figure S1**
